# Skeletal muscle and MASLD: Mechanistic and clinical insights

**DOI:** 10.1097/HC9.0000000000000711

**Published:** 2025-05-23

**Authors:** Thomas Marjot, Matthew J. Armstrong, Jonathan G. Stine

**Affiliations:** 1 Oxford Centre for Diabetes, Endocrinology and Metabolism (OCDEM), Radcliffe Department of Medicine, Churchill Hospital, University of Oxford, Oxford, UK; 2 Translational Gastroenterology and Liver Unit (TGLU), Nuffield Department of Medicine, John Radcliffe Hospital, University of Oxford, Oxford, UK; 3 Liver Unit, Queen Elizabeth University Hospital Birmingham, Birmingham, UK; 4 Birmingham NIHR Biomedical Research Centre, University of Birmingham, Birmingham, UK; 5 Department of Medicine, Division of Gastroenterology and Hepatology, Penn State Health—Milton S. Hershey Medical Centre, Hershey, Pennsylvania, USA

**Keywords:** adipose, exercise, hepatokines, insulin resistance, MASH, myokines, myosteatosis, obesity, sarcopenia

## Abstract

Metabolic dysfunction–associated steatotic liver disease (MASLD) is intrinsically linked with widespread metabolic perturbations, including within skeletal muscle. Indeed, MASLD is associated with a range of skeletal muscle abnormalities, including insulin resistance, myosteatosis, and sarcopenia, which all converge on the liver to drive disease progression and adverse patient outcomes. This review explores the mechanistic links between skeletal muscle and MASLD, including the role of abnormal glycemic control, systemic inflammation, and disordered myokine signaling. In turn, we discuss how intrinsic liver pathology can feed back to further exacerbate poor skeletal muscle health. Given the central importance of skeletal muscle in MASLD pathogenesis, it offers clinicians an opportunity to intervene for therapeutic benefit. We, therefore, summarize the role of nutrition and physical activity on skeletal muscle mass, quality, and metabolic function and discuss the knock-on effect this has on the liver. An awareness of these treatment strategies is particularly important in the era of effective pharmacological and surgical weight loss interventions, which can be associated with the development of sarcopenia. Finally, we highlight a number of promising drug agents in the clinical trial pipeline that specifically target skeletal muscle in an attempt to improve metabolic and physical functioning.

## INTRODUCTION

Metabolic dysfunction–associated steatotic liver disease (MASLD) is the most common chronic liver disease (CLD) worldwide and accounts for a huge global burden of morbidity and mortality.^1^ Since the original histological descriptions of the condition over 40 years ago, our fundamental understanding of disease pathogenesis has shifted enormously.^2^ A central component of this evolution has been the recognition that MASLD is a pervasive and multisystemic disease. This is exemplified by cardiovascular events and extrahepatic malignancy being the most common causes of mortality in patients with MASLD.^3^ There has also been a wider appreciation of the pathogenic role of other systemic disease states (eg, obesity and type 2 diabetes [T2D]) as well as organ-specific pathology, including adipose and skeletal muscle dysfunction.^4^ The fact that MASLD is intrinsically linked with widespread metabolic perturbations is also partly responsible for the nomenclature change away from NAFLD in 2023.^5^ Skeletal muscle is a highly active metabolic organ with a pivotal role in glucose control and its energy response to fasting and exercise.^6^ It also fine-tunes systemic, including hepatic, exposure to myokines and inflammatory mediators.^7^ However, in the setting of MASLD, skeletal muscle undergoes major physiological alterations culminating in insulin resistance, myosteatosis, and sarcopenia, which collectively increase the risk of physical frailty and disability. This review aims to discuss the reciprocal interaction between muscle and liver pathology with a focus on molecular mechanisms, clinical implications, and potential therapeutic strategies, including the role of exercise.

## SKELETAL MUSCLE METABOLIC DYSFUNCTION AND INSULIN RESISTANCE IN MASLD

### Measuring skeletal muscle insulin resistance in vivo

**FIGURE 1:**
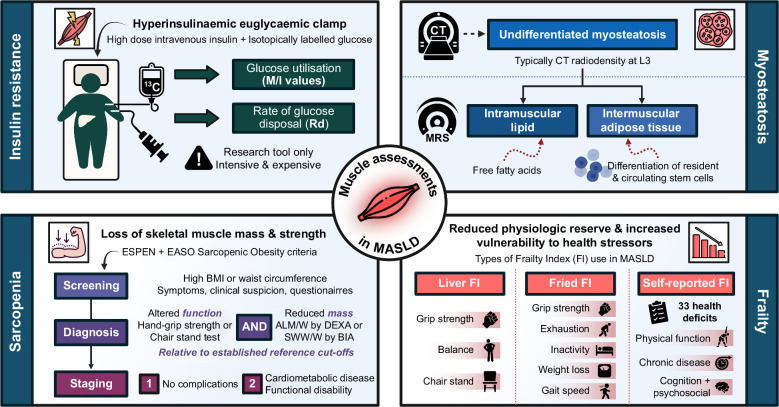
Diagnostic and assessment tools for muscle pathology in patients with MASLD. Figure created using biorender.com. Abbreviations: ALM/W, appendicular lean mass adjusted by weight; BIA, bioelectrical impedance analysis; BMI, body mass index; DEXA, dual-energy X-ray absorptiometry; EASO, European Association for the Study of Obesity; ESPEN, European Society for Clinical Nutrition and Metabolism; FI, frailty index; MASLD, metabolic dysfunction–associated steatotic liver disease; M/I value, glucose metabolism per unit of insulin; Rd, rate of glucose disposal; SWW/W, total skeletal muscle mass adjusted by weight.

Under normal physiological conditions, skeletal muscle is a highly insulin-responsive organ and is responsible for over 80% of all insulin-mediated glucose disposal. The gold standard method for assessing insulin resistance in vivo is widely regarded to be the 2-step hyperinsulinemic euglycemic clamp (Figure 1).^8^ First developed by DeFronzo et al^9^ in 1979, this procedure typically involves 2 sequential phases of i.v. insulin (low- and high-dose concentrations) alongside a simultaneous variable rate glucose infusion to maintain euglycemia at fasting basal levels. The glucose infusion rate is, therefore, highly indicative of insulin sensitivity, with enhanced insulin action associated with greater glucose requirements. The “low-dose” insulin phase of the clamp is designed to suppress endogenous (principally hepatic) glucose production (EGP), whereas “high-dose” insulin should switch off EGP completely and provide a robust stimulus for glucose disposal primarily by skeletal muscle. When the 2-step clamp is combined with the use of isotopically labeled glucose, these rates of EGP and glucose disposal can be calculated, which act as reliable surrogates for hepatic and peripheral insulin sensitivity, respectively. Diminished glucose disposal is recognized as an early harbinger of metabolic disease, being present decades before the evolution of pancreatic beta-cell failure, hyperglycemia, and T2D.^10^ As a result, it has even been suggested that skeletal muscle insulin resistance represents the primary defect in the pathogenesis of T2D.^11^ Clamp studies have shown that patients with MASLD have a reduction in rates of glucose disposal compared with healthy volunteers. For example, Bugianesi et al^12^ showed that peripheral disposal was 45% lower in nonobese, nondiabetic patients with MASLD compared with a well-matched healthy control cohort, even after adjusting for lean body mass. Similarly, a second study in North America used hyperinsulinemic euglycemic clamps to phenotype 190 members of the general population separated into groups with simple steatosis, metabolic dysfunction–associated steatohepatitis(MASH), or normal liver defined by histology and hepatic magnetic resonance spectroscopy.^13^ Those with simple steatosis and MASH had 50% and 60% reductions in glucose disposal, respectively, although there were imbalances in the prevalence of obesity and T2D between groups. In both these studies, MASLD-associated reductions in disposal were found to be far greater than the changes observed in EGP. This is further supported by the degree of steatosis and severity of MASH correlating more strongly with indices of peripheral compared to hepatic insulin resistance.^14^ This suggests that despite strictly being an intrinsic liver disease, MASLD appears to have a stronger association with skeletal muscle compared to hepatic insulin resistance. Indeed, the archetypal progression from MASLD to T2D develops only when hepatic autoregulation is lost and glucose production exceeds the capacity of muscle glucose disposal.^15^

### Drivers of skeletal muscle insulin resistance in MASLD

#### Adipose-muscle cross-talk

**FIGURE 2:**
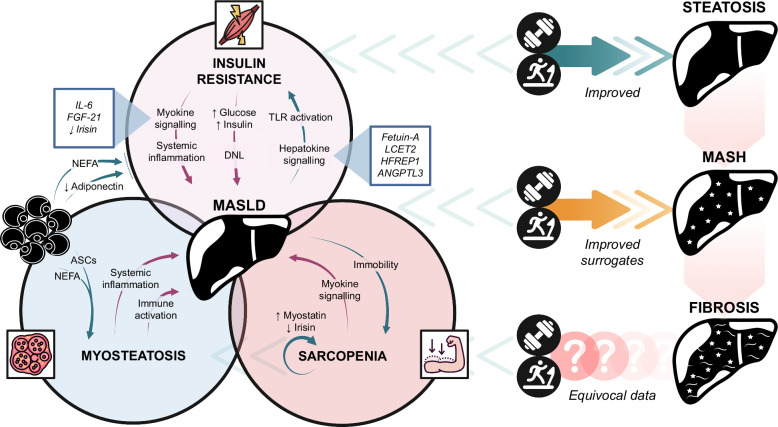
Bidirectional relationship between MASLD and skeletal muscle and the impact of exercise on pathogenic pathways and liver histology. Skeletal muscle insulin resistance, myosteatosis, and sarcopenia all converge on the liver to drive the progression of MASLD. Reciprocally, multiple pathways feed back from the liver to influence the function, quality, and quantity of skeletal muscle. Both aerobic and resistance exercises have widespread benefits in all 3 domains of muscle pathology. Exercise is also able to improve steatosis and reduce surrogate markers of fibroinflammation. Further rigorous, well-controlled clinical trials are required to definitively establish the effect of exercise on liver fibrosis. Abbreviations: ANGPTL3, angiopoietin-like protein 3; ASCs, adipose stromal cells; DNL, de novo lipogenesis; HFREP1, hepatocyte-derived fibrinogen-related protein 1; LCET2, leukocyte cell–derived chemotaxin 2; MASH, metabolic dysfunction–associated steatohepatitis; MASLD, metabolic dysfunction–associated steatotic liver disease; NEFA, nonesterified fatty acids; TLR, toll-like receptor.

The relationship between MASLD and skeletal muscle insulin resistance appears to be partly mediated through adipose tissue dysfunction and the concept of lipotoxicity (Figure 2).^16^ It has long been recognized that high concentrations of circulating nonesterified free fatty acids (NEFA), induced either through lipid-rich meals or lipid infusions, result in a reduction in insulin-stimulated glucose disposal.^17^ Subsequent work combining clamp studies and muscle magnetic resonance spectroscopy indicates that this results from NEFA-related inhibition of glucose transport, which is compounded by a >50% reduction in both the rate of muscle glycogen synthesis and glucose oxidation.^18^,^19^ This is corroborated by a range of preclinical and human data showing that elevated diacylglycerol levels in skeletal muscle lead to enhanced PKC signaling and reduced activation of IRS-1/Akt pathways.^20^ Further data in cultured myocytes also show that NEFA disrupts intracellular insulin signaling by activating toll-like receptor-mediated inflammatory pathways.^21^,^22^ Elevated NEFA is well-recognized in patients with MASLD who have enhanced rates of adipose lipolysis.^23^,^24^ This seems to be related not only to generalized adiposity but also to adipose dysfunction with plasma insulin failing to suppress both systemic and local (interstitial) concentrations of NEFA in patients with biopsy-proven MASH.^25^ Using thiazolidinediones to improve adipose tissue insulin sensitivity and thus reduce circulating NEFA has also been shown to have a knock-on effect on skeletal muscle insulin sensitivity.^26^ Lastly, elevated NEFA has been shown to have a strong negative correlation with peripheral glucose disposal, specifically in patients with MASLD, thus completing the cyclical cross-talk between adipose, skeletal muscle, and liver tissue. Metabolic flexibility and inflexibility are additional important concepts to consider. Metabolic flexibility refers to an individual’s capacity to physiologically switch from predominantly NEFA uptake and lipid oxidation during fasting to suppression of lipid oxidation and increased glucose uptake, oxidation, and storage under insulin-stimulated conditions.^27^ MASLD is characterized by metabolic inflexibility in which NEFA oxidative metabolism in skeletal muscle remains elevated and is not shifted toward glucose utilization with insulin.^15^,^28^ While this may initially be viewed as a hepatoprotective mechanism by limiting hepatic NEFA exposure, ultimately, the reduced glucose disposal propagates hyperglycemia and T2D, which then drives further liver steatosis and inflammation.^15^ Skeletal muscle insulin sensitivity may also be influenced in MASLD by systemic inflammation and immune activation, as well as through interactions with circulating adipokines. Increasing evidence suggests that obesity-related inflammation can manifest as increased skeletal muscle immune cell infiltration.^29^,^30^ For example, in both obesity and during stimulation with inflammatory molecules, myocytes upregulate chemokine secretion (eg, MCP-1), which induces immune cell migration in parallel with accumulating insulin resistance.^31^,^32^ Finally, adipokine adiponectin has key roles in muscle metabolic physiology, including pleomorphic anti-inflammatory and antifibrotic properties.^33^,^34^ It also has potent anti-lipotoxic effects by enhancing fatty acid oxidation as well as promoting whole-body insulin sensitivity and β-cell insulin secretion.^33^,^35^ Serum levels of adiponectin are known to be reduced in a range of metabolic conditions, including obesity, T2D, and MASH.^33^,^36^,^37^,^38^ Importantly, adiponectin has been shown to correct high-fat diet–induced disturbances in rodent muscle metabolomic profile with improvements in insulin-mediated glucose disposal,^39^,^40^ thereby suggesting that skeletal muscle may be a key intermediary between insulin resistance and adiponectin deficiency.^40^

#### Liver-muscle cross-talk and the role of hepatokines

Given the multisystemic influence of hepatic steatosis, there has been considerable interest in the liver as an endocrine structure that is able to participate in hepatokine-related organ cross-talk (Figure 2).^41^ Fetuin-A was the first major hepatokine to be described in detail. The hepatic expression of this protein is significantly elevated in patients with MASLD, and in rodent models, it is able to induce inflammatory signaling through TLR4, leading to skeletal muscle insulin resistance.^42^,^43^,^44^ Since these early findings, over 20 additional hepatokines have since been described with a potential pathogenic role in MASLD, many of which exert a negative effect on muscle metabolic physiology.^41^ For example, leukocyte cell–derived chemotaxin 2 is an energy-sensing hepatokine that is elevated in MASH and is able to impair myocyte insulin signaling through c-Jun NH2-terminal kinase phosphorylation.^45^,^46^ Similarly, serum levels of the hepatocyte-derived fibrinogen-related protein 1 (also known as hepassocin) are increased in MASLD, correlate with fasting glycemia, and appear to promote skeletal muscle insulin resistance through EGFR/c-Jun NH2-terminal kinase–mediated pathways.^47^,^48^ Finally, angiopoietin-like protein 3 increases in parallel with the severity of liver histology in patients with MASLD,^49^ and angiopoietin-like protein 3 inhibition is associated with a beneficial effect on whole-body insulin resistance and hepatic steatosis in mice.^50^ While all these circulating mediators offer an elegant biological link between liver and muscle pathology, their role has predominantly been established in preclinical models, and their impact and translational relevance are yet to be determined in real-world patients with MASLD.

### Reciprocal impact of skeletal muscle insulin resistance on the liver

There are several mechanisms through which skeletal muscle insulin resistance and impaired glucose disposal may feed back to promote the development and progression of MASLD. First, as discussed, reduced glucose disposal will ultimately precipitate hyperglycemia and hyperinsulinemia, which represents a highly lipogenic combination. Increased hepatic glucose availability provides a substrate for de novo lipogenesis (DNL), which is augmented by high insulin availability. Insulin promotes lipogenesis through the transcription and activation of sterol regulatory element–binding protein-1c, a master regulator of lipogenesis, and even in insulin-resistant conditions, insulin continues to selectively support DNL while failing to reduce hepatic glucose output.^51^,^52^,^53^,^54^ Deciphering the precise mechanisms for this differential regulation of hepatic lipid and glucose metabolism and quantifying its contribution to MASLD has proven a perpetual challenge and a point of contention within metabolic research.^4^,^55^,^56^ Nonetheless, clinical studies in patients with MASLD have clearly shown that hepatic DNL inversely correlates with whole-body insulin sensitivity and directly correlates with 24-hour plasma glucose and insulin concentrations.^57^ In addition, myokine alterations secondary to insulin resistance may negatively impact liver histology and metabolic homeostasis.^58^ For example, irisin, an exercise-induced myokine, appears to improve hepatic oxidative stress and inhibit fibrogenesis through suppression of HSC activation. Irisin is known to be downregulated in skeletal muscle across a range of insulin-resistant states, including obesity, T2D, and MASLD.^59^,^60^ Several myokines, including IL-6 and FGF-21, are known to be elevated in the context of insulin resistance. Levels of intrahepatic IL-6, a major proinflammatory cytokine, are increased in patients with MASLD, and sustained upregulation of IL-6 in rodent models is able to perpetuate hepatic insulin resistance.^61^,^62^ In contrast, under physiologic conditions, myocytic FGF-21 confers hepatoprotective effects through widespread insulin sensitization, diminished immune cell infiltration, and the upregulation of hepatic programs involved in fatty acid oxidation.^63^,^64^ In light of these positive downstream influences, the presence of elevated FGF-21 across a range of metabolic diseases suggests that MASLD may represent a state of relative FGF-21 resistance.^65^,^66^ More work is needed, particularly in clinical studies, to fully establish the role of myokines in human MASLD and to understand how the muscle-liver axis can be manipulated for therapeutic benefit.

## MYOSTEATOSIS AND MASLD

### Definition and diagnosis of myosteatosis

Myosteatosis refers to the presence of ectopic fat within skeletal muscle tissue and encompasses 3 anatomical patterns of lipid deposition (Figure 1).^67^ First, fat can accumulate beneath the muscle fascia in-between muscle groups as so-called intramuscular adipose tissue (IMAT); second, it can be found within the muscle interstitium as extramyocellular lipid; and lastly, triglyceride can collect as intramyocellular lipid content (IMLC) to be directly utilized as metabolic fuel.^68^ While the term myosteatosis refers to all 3 fat distributions, quantifying lipid content according to specific location depends on the diagnostic modality.^69^ CT remains the most widely utilized method of identifying myosteatosis, and although it highlights lipid as areas of lower mean muscle radiodensity, it is unable to discriminate the microscopic location of ectopic fat. Typical anatomical locations for myosteatosis measurements using CT include the trunk at the level of the L3, proximal femur, and cervical vertebrae. Similarly, thigh ultrasound imaging only allows for semi-quantitative measurements of myosteatotic burden, albeit there is no radiation exposure, and it can be performed in outpatients. In contrast, ^1^H-magnetic resonance spectroscopy is able to measure relative contributions of intra- and extramyocellular lipid content by leveraging different frequencies of hydrogen excitation. It is important to note that conventional measures of muscle mass (ie, bioelectrical impedance analysis [BIA], dual-energy X-ray absorptiometry [DEXA]) cannot identify myosteatosis. While muscle biopsies are not feasible in routine clinical care, a small number of research studies in obese participants have shown the presence of histological IMLC to be associated with muscle inflammatory infiltrates, MASH, and advanced hepatic fibrosis.^70^,^71^

### Prevalence and prognostic significance of myosteatosis in MASLD

The true prevalence of myosteatosis in MASLD remains poorly understood owing to significant study heterogeneity and lack of standardization in imaging modality used, muscle groups assessed, and the method of MASLD identification.^72^,^73^,^74^ Nonetheless, there is consensus that myosteatosis is both common and substantially more prevalent than sarcopenia in patients with MASLD. Using UK population data, for example, it was estimated that 24% of patients with MASLD have isolated myosteatosis, 10% have sarcopenia, and 14% have both muscle conditions simultaneously.^75^ This latter group with coexisting myosteatosis and sarcopenia was found to have double the risk of poor functional performance and triple the prevalence of T2D and CHD compared to those without comorbid muscle disease.^75^ It is also notable in one study that 31% of nonobese patients with MASLD exhibit myosteatosis on abdominal CT, suggesting that this may be a unique and underrecognized contributor to lean MASLD and MASH.^76^ Myosteatosis is now well-established as an independent predictor of both baseline liver disease severity and fibrosis progression assessed by interval transient elastography.^77^,^78^,^79^ Furthermore, a single longitudinal study has identified myosteatosis as a strong predictor of all-cause mortality in MASLD,^75^ which aligns with larger data sets showing that high muscle fat content is independently associated with all-cause mortality in the general population.^80^ Lastly, myosteatosis is extremely common in cirrhosis populations, with a pooled prevalence of 46%,^81^ and is associated with higher MELD,^82^ Child-Pugh scores,^81^,^82^,^83^ coexisting sarcopenia,^82^ incident overt HE,^84^ and overall survival.^82^,^84^,^85^,^86^,^87^ However, meta-analyses have shown that the prevalence of myosteatosis in cirrhosis is significantly higher in studies using body mass index (BMI) (56%) compared with gender-specific adjustments (36%).^81^ This further reinforces the need to establish and validate optimal criteria for assessing myosteatosis in the context of liver disease before this important parameter can be widely integrated into clinical risk stratification.^88^

### Pathological mechanisms linking myosteatosis with liver disease progression

Although most observational studies have tended to report on global myosteatosis, it is worth considering the regulation and balance between IMAT and IMLC as it offers further mechanistic insights into the muscle-liver axis. IMAT can generally be viewed as a spill-over of genuine adipose tissue into the muscle compartment and is therefore positively associated with BMI and visceral or subcutaneous adipose tissue depots.^89^,^90^,^91^ Influential work in preclinical models has shown that multipotent adipose stromal cells can be released into circulation from subcutaneous adipose tissue, after which they take up residence in skeletal muscle under the control of CXCL12 and its receptor CXCR4.^92^ Furthermore, overnutrition can promote the mobilization of subcutaneous adipose stromal cells leading to expansion of IMAT.^93^ IMAT has consistently been shown to correlate with reduced peripheral insulin sensitivity measured through hyperinsulinemic euglycemic clamp studies,^91^,^94^,^95^ glucose tolerance testing,^96^ fasting plasma glucose levels,^97^ and HOMA-IR.^97^ Importantly, many of these observed associations remain significant after adjusting for BMI, suggesting that the presence of IMAT has an independent effect on metabolic health beyond simply reflecting generalized adiposity.^95^,^98^ A range of studies have also demonstrated that IMAT is associated with T2D^96^ and multiple cardiovascular risk factors, including dyslipidemia, carotid artery intima-media thickness, and coronary artery calcification.^99^,^100^,^101^ The effect of IMAT on adjacent muscle function remains an area of active ongoing investigation. Studies have suggested that the presence of IMAT may impinge on muscle insulin sensitivity through a combination of impaired blood flow, direct lipotoxicity, and the deleterious effects of proinflammatory adipokines.^102^,^103^ Alongside altering metabolic phenotype, this milieu may promote further IMAT expansion by facilitating the differentiation of resident fibro-adipogenic progenitor cells into adipocytes.^104^ IMAT may also contribute to the chronic systemic inflammation associated with obesity, MASLD, and increased cardiovascular risk, with studies showing that IMAT is positively associated with plasma concentrations of IL-6 and CRP after controlling for important cofactors, including BMI and total muscle mass.^105^ IMAT should, therefore, be regarded as a discreet, organized, and metabolically active entity, which involves cross-talk between multiple cell types, including adipocytes, fibroblasts, endothelial, and immune cells.^106^,^107^ Indeed, multi-omics approaches in rodent models have indicated that IMAT has a distinct set of metabolic and secretory properties compared with subcutaneous and visceral adipose tissue.^106^,^108^ IMAT also appears to physically disrupt neuromuscular activation and muscle fiber alignment, leading to sarcopenia and impaired muscle strength.^109^,^110^ Taken together, a conceptual framework can be established whereby overnutrition leads to IMAT accumulation and impaired myocyte function, which in turn drives MASLD through systemic inflammation, dyslipidemia, and impaired glucose tolerance. However, there is a notable lack of detailed in vivo studies that describe the direct relationship between myosteatosis and functional metabolic pathways involved in hepatic lipid flux, including hepatic insulin resistance and DNL.

Compared with IMAT, the pathogenic role of IMLC is more ambiguous. A number of studies have demonstrated that excess IMLC is associated with insulin resistance and T2D. However, to what extent IMLC represents a cause or effect of systemic insulin resistance has remained challenging to elucidate. It is plausible that intramyocellular triglyceride is derived from the esterification of excess circulating free fatty acids, in the same way that a majority of intrahepatic lipid in MASLD is derived from adipose lipolysis.^111^ Furthermore, in vitro work has suggested that localized lipolysis of IMAT may flood adjacent myocytes with diacylglycerol, which then has a knock-on effect on insulin signaling.^112^ However, it has always been difficult to reconcile these observations with the fact that IMLC is increased in highly insulin-sensitive endurance-trained individuals (known as the “athlete’s paradox”).^113^ This would suggest that IMCL accumulation per se is not a causal factor in skeletal muscle insulin resistance. Introducing further complexity is the fact that physical exercise in patients with MASLD has been shown to result in ILMC reductions with corresponding improvements in insulin sensitivity, independent of changes in body weight and hepatic steatosis.^114^ Taken together, the role and impact of IMLC appear to be highly context-dependent, being deleterious in the setting of metabolic risk factors and advantageous in high-performance athletes. There is also contention regarding the role of mitochondrial function in IMLC. Some studies have indicated that diminished mitochondrial oxidative capacity in skeletal muscle may be a primary defect in the pathogenesis of insulin resistance by promoting IMLC expansion.^115^ However, further work in patients with MASLD has failed to demonstrate clear associations between ILMC, liver fat fraction, and surrogate markers of mitochondrial function.^116^

## SARCOPENIA AND MASLD

### Conceptual and practical definitions of sarcopenia and frailty

Sarcopenia is characterized by progressive and generalized loss of skeletal muscle mass with associated decline in muscle strength and function. This phenomenon is best recognized as part of aging, but it can occur earlier in life, and there has been a renewed emphasis on sarcopenia as an important component of obesity, metabolic disease, MASLD, and advanced CLD. In 2016, sarcopenia was finally recognized as a disease state in ICD-10 (code M62.84), despite being acknowledged as a clinically important entity since the 1980s.^117^ However, arriving at a consensus classification of such a complex condition has proved challenging. In 2024, a global initiative arrived at an internationally accepted conceptual definition that described 3 central characteristics of sarcopenia: loss of muscle mass, impaired muscle strength, and reduced muscle-specific strength (eg, the ratio of muscle strength to muscle size).^118^ Importantly, this conceptual definition should not need adjusting according to age or clinical picture (eg, CLD, heart failure, chronic kidney disease, or cancer). While this framework has unified our broad understanding of what sarcopenia represents, there is now an urgent need to develop a practical, operational definition for use in both clinical and research settings. Currently, most studies (including in the context of CLD) have tended to use the approach set out in the revised 2019 European consensus criteria.^119^ This permits the use of a range of tests for muscle strength (eg, hand grip strength [HGS], chair stand test) and mass (eg, through BIA or DEXA), with probable sarcopenia defined when parameters fall >2 SDs below a relevant mean reference value.^119^,^120^,^121^ In addition, the European Society for Clinical Nutrition and Metabolism (ESPEN) and the European Association for the Study of Obesity (EASO) have set out a structured procedure for the diagnosis of sarcopenic obesity (Figure 1). Sarcopenic obesity is a clinical and functional condition characterized by the coexistence of obesity, excess fat mass, and sarcopenia, which affects 6%–10% of individuals with a BMI >30 kg/m^2^ and has consistently been shown to be associated with adverse metabolic outcomes.^122^,^123^ This ESPEN-EASO approach is endorsed by the European Association for the Study of the Liver (EASL), and it is reasonable to apply this framework to obese patients with coexistent MASLD^124^ (Figure 1). It is worth noting that a single liver-specific sarcopenia scoring system exists, which was developed by the Japanese Society of Hepatology in 2015. This includes disease-specific cutoff values (eg, for BIA and DEXA) but was developed using a predominantly decompensated cirrhosis cohort with chronic viral hepatitis, which has limited generalizability and widespread use.^125^ Cross-sectional imaging, with CT or MRI, is able to offer accurate, reproducible, and objective measures of muscle mass and quality in multiple muscle groups.^126^ Unlike BIA and DEXA, these modalities are not affected by tissue edema or extremes of BMI. The lumbar 3 skeletal muscle index, which provides a measurement of whole-body skeletal muscle volume normalized to body surface area, has enabled specific diagnostic cutoffs of sarcopenia to be developed in patients with end-stage liver disease^127^ However, serial measures should be avoided due to radiation exposure and health care costs and there has been no specific validation in MASLD cohorts. Despite all these attempts at standardization, a vast array of different tests for sarcopenia continue to be employed in clinical and academic settings, many of which exhibit geographical variability or are confounded by demographic and anthropomorphic variables. This heterogeneity has made it difficult to accurately assess disease prevalence and has hampered therapeutic research and the development of clinical care pathways.^118^

Finally, frailty is a term that has become increasingly employed across a range of clinical settings to describe a complex process of reduced physiologic reserve and increased vulnerability to health stressors, in which sarcopenia plays a contributory role.^128^ A number of physical frailty scoring systems are available, which often include overlapping assessments with sarcopenia (eg, HGS) (Figure 1).^128^ Within MASLD populations, the liver frailty index, Fried frailty index, and self-reported frailty index have all been employed^129^,^130^ (Figure 1). A recent meta-analysis in 2025 of 18 studies estimated the pooled prevalence of physical frailty in individuals with MASLD as between 8% and 23%, with patients having double the risk compared to non-MASLD comparator cohorts.^129^ Physical frailty in the UK general population also appears to be associated with incident CLD, including MASLD, cirrhosis, liver cancer, as well as liver-related mortality.^131^

### Epidemiology of sarcopenia in MASLD

Despite variability in diagnostic approach, sarcopenia is a well-recognized phenomenon even in noncirrhotic MASLD and MASH.^122^ A number of interval meta-analyses of observational studies have shown an increased risk of sarcopenia in individuals diagnosed with MASLD compared with those without.^132^,^133^,^134^,^135^ Most recently, pooled analysis of 25 studies showed that MASLD was significantly associated with sarcopenia (OR=1.25), although odds varied according to adjustment by height, weight, or BMI.^132^ The point prevalence of sarcopenia in MASLD populations also varied, ranging from 10% to 70%, but appears to be comparatively higher than in other CLD etiologies.^132^ Indeed, a US study found that sarcopenia was most common in patients with MASLD (41%), followed by ALD (27%), HCV (22%), HBV (17%), and non-CLD controls (19%).^121^ Importantly, a number of studies have highlighted that the presence of sarcopenia is associated with evidence of hepatic fibrosis defined through noninvasive tools. For example, cross-sectional data from NHANES showed that advanced fibrosis (defined by NAFLD fibrosis score) was more common in patients with MASLD with sarcopenia (defined through BIA) compared with those without (7.8 vs. 1.6%; *p*<0.001).^136^ Similarly, nationwide survey data from Korea showed that sarcopenia (defined using DEXA) conferred an increased risk of advanced fibrosis (defined by BARD and FIB-4). A number of studies have shown that this increased risk of advanced fibrosis in sarcopenic patients with MASLD remains significant after controlling for other metabolic and liver risk factors, including physical activity, smoking status, and T2D.^136^ Lastly, longitudinal US data from patients with MASLD has shown that baseline sarcopenia is able to predict cardiovascular and liver-related mortality over >25-years of follow-up.^121^ This impact on clinical outcomes in patients with MASLD is further supported by UK Biobank data, indicating that low muscle mass and HGS are highly predictive of incident hospitalization and death.^137^

### Mechanistic basis for sarcopenia in MASLD and obesity

The mechanistic basis of sarcopenia has a significant overlap with the dominant pathways driving wider metabolic dysfunction, including MASLD. Namely, muscle mass and function are negatively influenced by insulin resistance, systemic inflammation and oxidative stress, adipose lipolysis/lipotoxicity, and dysregulated organokine signaling.^122^ In a cohort of patients with a high burden of cardiometabolic comorbidities, CRP and IL-6 were found to be positively associated with total fat mass and negatively associated with lean mass.^138^ Similarly, a number of studies have shown that markers of insulin resistance (eg, HOMA-IR) are associated with increased sarcopenia risk.^139^,^140^ Muscle homeostasis relies on maintaining a careful balance of anabolic and catabolic myokines.^141^ Broadly, myostatin acts as a catabolic signal and serves to dampen myocyte proliferation, whereas irisin functions to promote the expansion of muscle tissue. During the natural aging process, there is a shift in the ratio of myostatin to irisin, which contributes to age-related sarcopenia.^142^,^143^ Similar patterns are observed in patients with MASLD, obesity, and cirrhosis.^144^,^145^,^146^,^147^ Lastly, sarcopenia is accelerated by immobility, and many patients with obesity and MASLD will have reduced physical functioning due to osteoarthritis and cardiorespiratory comorbidities. Fortunately, even chair-based exercises are able to counter the loss of muscle function with associated metabolic benefits.^148^,^149^,^150^

### Risk of sarcopenia with weight loss therapies

Weight loss remains a cornerstone in the management of MASLD and is linked to improvements in both liver and cardiometabolic outcomes.^151^ As a result, pharmacological weight loss (eg, with glucagon-like peptide 1 receptor agonists [GLP-1RAs] ± gastric inhibitory polypeptide analogs) and bariatric surgery are now widely utilized in the management of metabolic conditions in patients with MASLD. However, there have always been concerns that these interventions may precipitate loss of lean muscle mass, which could offset any improvements in metabolic health related to decreased fat mass. Indeed, while GLP-1RAs are associated with a mean total body weight reduction of 10%–20%, up to 40% of this weight loss can be accounted for by a decline in skeletal muscle mass.^152^,^153^ This exceeds the 20% contribution of lean mass to total weight loss observed with calorie-restrictive diets.^154^ For context, the decline in muscle mass with GLP-1RA is several times greater than that would be expected from typical age-related muscle loss (1% per year)^154^ and even exceeds muscle loss associated with chemotherapy regimens and some advanced cancers.^153^ As a result, in T2D populations, the use of GLP-1RA has been associated with reduced HGS and a higher incidence of falls compared with untreated patients.^155^ In addition, weight regain after GLP-1RA discontinuation is predominantly accounted for by a rebound in fat mass rather than muscle mass, and, therefore, repeated cycles of weight instability are linked to a higher risk of sarcopenic obesity. The effect of bariatric surgery on muscle mass appears to be less profound but is still associated with 23% of weight loss at 12 months being accounted for by reductions in lean body mass.^156^ Fortunately, protein-based nutrition and resistance exercise therapy offer a promising strategy to preserve muscle mass when delivered alongside weight loss interventions.^157^,^158^ Even though it is poorly studied in MASLD, it is essential that individuals meet their dietary protein targets (eg, 1.0–1.5 g/kg of ideal body weight/d) in conjunction with initiating pharmacological weight loss to preserve muscle mass. A number of randomized trials have shown that resistance training can minimize muscle loss while preserving total weight loss in obese adults undergoing dietary calorie restriction^159^,^160^ or bariatric surgery.^161^ Although aerobic exercise programs appear to act synergistically with GLP-1RA to help improve skeletal muscle mass and insulin sensitivity,^162^,^163^ the impact of weight-neutral resistance exercise on body composition remains to be investigated.

### Future pharmacological agents for the treatment of sarcopenia

There are currently no licensed medications for sarcopenia, and drug development has been challenging due to the complex and heterogenous nature of the disease. Optimizing nutrition (ie, protein targets) remains the mainstay for reversing sarcopenia in MASLD, with most of the international guidance focusing on nonobese cohorts and advanced CLD.^164^ The precise caloric target to improve muscle mass/strength while losing adipose remains unresolved, with data specifically lacking in sarcopenic obesity and MASLD. However, various drug agents have shown promise.^165^ Testosterone supplementation has the largest evidence base and has a favorable efficacy and safety profile in older adults when delivered at physiological concentrations, although the optimal dose and route of administration remain unclear.^166^,^167^,^168^ Randomized control data have shown that intramuscular testosterone leads to improvements in muscle mass at 1 year in men with cirrhosis and low serum testosterone.^169^ Similarly, a phase II study of the novel androgen receptor agonist LPCN 1148 also improved sarcopenia and reduced episodes of overt HE in a mixed cohort of patients with advanced cirrhosis (21% MASH).^170^ Androgen deficiency has been linked with steatosis and increased hepatic DNL, and another phase II study is currently in progress investigating the impact of testosterone analogs on hepatic fat fraction and appendicular lean muscle mass in patients with noncirrhotic MASH.^171^ Vitamin D deficiency may increase the risk of sarcopenia and has been separately associated with fibrosis progression in MASLD.^172^ Even though the literature is inconsistent in elderly populations, vitamin D replacement, especially in those with low serum 25(OH)D concentrations, warrants further research in sarcopenic obesity and MASLD. Similarly, beta-hydroxy-beta-methylbutyrate, which may enhance muscle protein synthesis by the mTOR pathway and production of IGF-1, warrants further investigation. To date, it has shown improvement in both muscle mass and function, although studies are significantly limited in size (n<50) and restricted to those with cirrhosis.^173^ Bimagrumab (BYM338) is a humanized monoclonal antibody that binds to the activin type II receptor and blocks its interaction with myostatin, a negative endogenous regulator of skeletal muscle growth. This has shown potential in sarcopenic obesity, where it serves to both increase muscle mass (with improvements in glucose disposal) and simultaneously decrease fat mass.^174^,^175^ Preclinical work has also started to explore the role of myostatin inhibition alongside GLP-1RA therapy to preserve muscle mass^176^ (abstract form only). Lastly, BIO101 has emerged as a potential candidate in recent years and is able to improve muscle function in older adults by stimulating myoblast differentiation.^177^ Despite the shifting landscape of potential pharmacological options for sarcopenia, as yet, there have been no published drug trials specifically in the context of MASLD (Box 1).

Box 1

**Table:** Areas of uncertainty and future directions

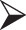 Establish internationally recognized practical diagnostic and assessment criteria for sarcopenia and myosteatosis
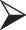 Characterize the impact of myosteatosis and sarcopenia on functional pathways governing intrahepatic lipid accumulation (eg, hepatic insulin resistance, DNL)
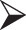 Understand the impact of exercise on myokine signaling in patients with MASLD
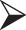 The role of exercise (and the optimal exercise training strategy) to improve histological and clinical outcomes in patients with MASLD
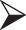 Efficacy and safety profile of novel pharmacological agents for sarcopenia in patients with MASLD
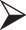 Impact of loss of muscle mass and sarcopenia on liver and cardiovascular outcomes in patients receiving weight loss interventions (eg, dietary calorie restriction, GLP-1RA therapy, bariatric surgery)
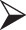 Determine optimal strategies to mitigate the loss of muscle mass in patients with MASLD receiving weight loss interventions
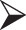 Methods to improve patient education about muscle health and nutrition, and promote engagement with physical activity/exercise training in MASLD cohorts

## THE BENEFITS OF EXERCISE IN MASLD

Physical activity is known to have a profound impact on many of the pathways discussed above, which converge on the liver to influence the pathogenesis of MASLD. In the field of exercise medicine, adopting precise definitions is particularly important as many of the terms in widespread use can be variably interpreted by patients and clinicians.^178^,^179^ Physical activity refers to any movement produced by skeletal muscle that requires energy expenditure. Exercise training is a subcategory of physical activity that is planned, structured, repetitive, and has the primary purpose of improving health or physical fitness. Aerobic exercise training describes activities that are moderate-intensity and sufficient to maintain or improve cardiorespiratory fitness. Conversely, resistance training refers to programs that increase the strength, power, and mass of major skeletal muscle groups. Aerobic training is generally regarded as a potential contributor to weight loss, whereas resistance training typically results in weight-neutral outcomes.^178^

The metabolic impact of exercise training cannot be overstated (Figure 2).^180^ Experimental studies in human participants show that even short durations of either aerobic or resistance exercise can improve insulin sensitivity and attenuate systemic NEFA concentrations.^181^,^182^,^183^,^184^,^185^,^186^ With aerobic regimens, these changes occur well before the onset of weight loss and body fat reduction. Indeed, hyperinsulinemic euglycemic clamp studies in patients with T2D have shown that even a single bout of exercise can improve hepatic and peripheral insulin resistance at 12 hours.^187^ This likely occurs through a combination of increased glucose transporter expression and improved insulin signal transduction.^188^ Both aerobic and resistance exercise training have also been shown to improve skeletal muscle mitochondrial number and function, reduce the systemic burden of inflammation, and improve myosteatosis in metabolic disease.^114^,^189^,^190^,^191^ Pooled analysis of studies has indicated that aerobic and combined training may increase physical function but not muscle mass in MASLD.^192^ However, results must be interpreted with caution due to small recruitment numbers and the complete absence of studies investigating resistance training alone, which has been proven to improve age-related sarcopenia.^193^ The weight loss–independent effects of exercise on liver and metabolic parameters are nicely demonstrated in MASLD populations. For example, Oh et al^194^ randomized 185 male Japanese patients with MASLD to receive either aerobic exercise therapy (90 min, 3 d a week) or continuous calorie restriction for 3 months.^194^ Despite losing less weight than dieting individuals, the exercise group still had significant improvements in liver function tests, hepatic steatosis (through controlled attenuation parameter values), liver stiffness measurements, serum NEFA concentrations, and markers of insulin resistance. A mechanistic substudy also showed a marked shift in organokine profiles, with exercise able to induce significant changes in adiponectin, myostatin, and leptin.^194^

There is now a considerable body of randomised controlled trials data evaluating the benefit of exercise regimens in MASLD (Table 1).^195^,^196^,^197^,^198^,^199^,^200^,^201^,^209^,^210^,^211^,^212^,^213^,^214^,^215^,^216^,^217^,^218^,^219^,^220^,^221^,^222^,^223^,^224^ Meta-analysis of studies using imaging biomarkers has previously shown that exercise therapy convincingly leads to a reduction of hepatic steatosis.^225^ Indeed, exercise interventions were >3.5 times more likely to achieve ≥30% relative reduction in MRI-measured liver fat fraction than those in control arms. A majority of these clinical trials have employed aerobic exercise regimens, and it has emerged that at least ≥750 metabolic equivalents of task minutes per week (eg, 150 min/wk of brisk walking) are required for treatment response.^225^ Fewer studies have utilized resistance training, but these have also shown improvements in steatosis and serum markers of liver and cardiometabolic health and may be more acceptable to patients with comorbidities limiting aerobic capacity.^197^,^209^ Although several studies have shown exercise-related reductions in surrogates of hepatic fibroinflammation,^204^,^226^,^227^ there is a relative paucity of data reporting on histological endpoints. A small number of exercise randomised controlled trials have shown improvements in fibrosis, hepatocyte ballooning, or NAS scores,^206^,^211^,^216^ although several were combined with dietary interventions,^206^,^211^ and other trials have failed to demonstrate any histological improvements.^228^ Translating clinical research into routine practice is also limited by heterogenous study populations, wide variability in the training programs delivered, and a lack of head-to-head comparisons between different exercise protocols. Nonetheless, the body of evidence reporting on the positive impact of exercise on metabolic outcomes, steatosis, and pathways involved in MASLD/MASH is considerable.^229^ Despite this, <25% of individuals with MASLD achieve guideline-based amounts of weekly physical activity.^230^,^231^ In addition to educating patients about the benefits of exercise, clinicians should consider offering personalized exercise prescriptions using FITT principles (Frequency, Intensity, Type, and Timeframe/duration)^229^,^232^,^233^ and focus on motivational/behavioral techniques to improve both engagement and compliance with exercise.

**TABLE 1:** Summary of major exercise intervention trials in MASLD

Study	Duration	Participants	Demographics	Supervision	Exercise frequency	Exercise intensity	Exercise session time	Exercise type	Weight loss	Adherence
Sullivan et al, USA^195^	16 wk	NAFLD with obesity (n=18) Exercise (n=12) Control (n=6)	Age: Exercise 48 y Control 49 y BMI: Exercise 37.1 kg/m^2^ Control 40.0 kg/m^2^ Male: Exercise 33% Control 17%	Yes	5 d/wk	Moderate (45%–55% VO_2_ peak)	30–60 min	Aerobic	NR	NR
Pugh et al, UK^196^	16 wk	NAFLD (n=11) Exercise (n=6) Control (n=5)	Age: Exercise 45 y Control 51 y BMI: Exercise 31.0 kg/m^2^ Control 30.0 kg/m^2^ Male: 54%	Yes	3–5 d/wk	Moderate (30%–60% MHR)	30–45 min	Aerobic	−2.2 kg (−2.4%)	NR
Hallsworth et al, UK^197^	12 wk	NAFLD (n=23) Exercise (n=12) Control (n=11)	Age: Exercise 54 y Control 52 y BMI: Exercise 31.0 kg/m^2^ Control 31.0 kg/m^2^ Male: NR	No	3 d/wk	Vigorous (75%–80% RM)	30–40 min	HIIT	−1.4 kg (−1.5%)	NR
Cuthbertson et al, UK^198^	16 wk	Sedentary NAFLD (n=50) Exercise (n=30) Control (n=20)	Age: Exercise 52 y Control 50 y BMI: Exercise 30.7 kg/m^2^ Control 29.7 kg/m^2^ Male: Exercise 77% Control 80%	Yes	3–5 d/wk	Moderate (30%–60% HRR)	30–45 min	Aerobic	−2.5 kg (−2.6%)	NR
Shojaee-Moradie et al, UK^199^	16 wk	Males with NAFLD (n=27) Exercise (n=15) Control (n=12)	Exercise 52 y Control 53 y BMI: Exercise 31.6 kg/m^2^ Control 31.7 kg/m^2^ Male: 100%	Yes	4–5 d/wk	Moderate (40%–60% HRR)	20–60 min	Aerobic	−4.0 kg (−3.8%)	NR
Cheng et al, China^200^	12 wk	NAFLD with impaired fasting glucose (n=40) Exercise (n=22) Control (n=18)	Age: Exercise 60 y Control 59 y BMI: Exercise 27.3 kg/m^2^ Control 27.1 kg/m^2^ Male: Exercise 21% Control 24%	Yes	2–3 d/wk	Moderate-vigorous (60%–75% VO_2_ peak)	30–60 min	Aerobic	−1.0 kg (−1.4%)	NR
Houghton et al, UK^201^	12 wk	Biopsy-proven NASH (n=24) Exercise (n=12) Control (n=12)	Exercise 52 y Control 51 y BMI: Exercise: 33.0 kg/m^2^ Control: 33.0 kg/m^2^ Male: NR	Yes	3 d/wk	Aerobic: Vigorous (90% MHR) RT: Vigorous (60%–70% RM)	45–60 min	Aerobic+RT	+1.0 kg (+1.1%)	NR
Brouwers et al, Netherlands^202^	12 wk	Sedentary NAFLD with obesity (n=22) Exercise (n=11) Control (n=11)	Age: Exercise 55 y Control 58 y BMI: Exercise 30.9 kg/m^2^ Control 29.5 kg/m^2^ Male: 100%	Yes	3 d/wk (2 d AT, 1 d RT)	Aerobic: Vigorous (70% Wmax) RT: 60% MVC	30 min	Aerobic+RT	Exercise −0.9 kg Control +0.25 kg	95% exercise session attendance
Abdelbasset et al, Egypt^203^	8 wk	NAFLD with diabetes and obesity (n=47) Exercise (n=31, 16 HIIT, 15 AT) Control (n=16)	Age: HIIT 54 y AT 55 y Control 55 y BMI: HIIT 36.3 kg/m^2^ AT 36.7 kg/m^2^ Control 35.9 kg/m^2^ Male: HIIT 63% AT 53% Control 56%	No	3 d/wk	Aerobic: Moderate (60%–70% VO_2_max) HIIT: Vigorous (80%–85% VO_2_max)	40–50 min	Aerobic HIIT	NR	NR
Stine et al, USA^204^	20 wk	Sedentary biopsy-proven NASH (n=28) Exercise (n=18) Control (n=10)	Exercise 53 y Control 45 y BMI: Exercise 34.3 kg/m^2^ Control 35.1 kg/m^2^ Male: Exercise 33% Control 50%	Yes	5 d/wk	Moderate (45%–55% VO_2_ peak)	45 min	Aerobic	−2.5 kg (−2.6%)	82% overall completion 89% exercise adherence (completion of ≥80% of exercise sessions)
Keating et al, Australia^205^	12 wk	NAFLD (n=14) Exercise (n=8) Control (n=6)	Exercise 53 y Control 61 y BMI: Exercise 39.6 kg/m^2^, Control 38.3 kg/m^2^ Male: Exercise 75%, Control 50%	Yes	3 d/wk	High (85%–95% max HR)	25 min	HIIT	NR	100% global adherence
Mucinski et al, USA^206^	10 mo	Sedentary biopsy-proven MASH (n=24) Exercise (n=16) Control (n=8)	Exercise 57 y Control 47 y BMI: Exercise 40.7 kg/m^2^ Control 37.5 kg/m^2^	Yes	3 d/wk	High (90%–95% max HR)	25 min	HIIT	Exercise −7.8 kg (−6.7%) Control −1.9 kg (−1.8%)	91% exercise sessions attended
Freer et al, Australia^207^	12 wk	Sedentary NAFLD (n=28) Exercise (n=14) Control (n=14)	Exercise 63 y Control 64 years BMI: Exercise 33.7 kg/m^2^ Control 31.1 kg/m^2^ Male: Exercise 14% Control 0%	Yes (remote with telehealth)	3 d/wk	Moderate (5–8 RPE)	25 min	AT (brisk walking)+RT	Exercise −2.5 kg (2.7%) Control −0.8 kg (−1.0%)	73% completed RT 52% completed AT
Us Altay et al, Turkey^208^	12 wk	NAFLD (n=62) Exercise (n=16) Exercise+Diet (n=14) Control (n=16)	Male: Exercise 25% Exercise+diet 29% Control 25%	No	5 d/wk	Not specified	30 min	Aerobic (walking)	Exercise −0.6 kg (0.0%) Diet+Exercise −1.5 kg (−1.6%) Control +0.3 kg (0.0%)	NR

Abbreviations: AT, aerobic training; BMI, body mass index; HIIT, high-intensity interval training; HRR, heart rate reserve; MHR, maximal heart rate; MVC, maximal voluntary contraction; MASH, metabolic dysfunction–associated steatohepatitis; MASLD, MASLD, metabolic dysfunction–associated steatotic liver disease; NAFLD, nonalcoholic fatty liver disease (original terminology used for studies pre-dating nomenclature change to MASLD and MASH); NASH, nonalcoholic steatohepatitis (original terminology used for studies pre-dating nomenclature change to MASLD and MASH); NR, not recorded; RM, repetition maximum; RPE, rating of perceived exertion; RT, resistance training; Wmax, maximal work capacity.

## CONCLUSIONS

Skeletal muscle has a crucial role to play in the pathogenesis of MASLD, with insulin resistance, myosteatosis, and sarcopenia all converging on the liver to drive disease progression. Indeed, in certain domains (eg, insulin resistance), there is a greater association between MASLD and metabolic dysfunction in skeletal muscle than there is within the liver itself. This highlights the need for a more integrated approach to the muscle-liver axis within both translational research and clinical practice. A range of tools are available to help assess muscle mass, quality, and function. Although many of these are imperfect, it is nonetheless critical for clinicians to recognize the importance of skeletal muscle health and attempt to include risk stratification within clinical care. This is particularly relevant in the era of effective weight loss interventions, including GLP-1RA and bariatric surgery, which are associated with loss of muscle mass alongside reduced adiposity. While several promising drug agents for sarcopenia move through late-phase clinical trials, nutrition (protein-based) and exercise therapy represent accessible and highly effective ways to improve multiple domains of skeletal muscle function with associated benefits on liver health.
